# Breaking the silos: a systematic review of oral health integration strategies for improved oral health and cardiovascular outcomes

**DOI:** 10.3389/fpubh.2026.1795955

**Published:** 2026-05-05

**Authors:** Wania Usmani, Charlotte Park, Sharta Ogato, Millicent Raymond, Maximilian Pangratius de Courten, Peter W. Lange, Fahad Hanna

**Affiliations:** 1 Department of Health and Education, Torrens University Australia, Melbourne, VIC, Australia; 2 Department of Public Health, Georgetown University, Washington, DC, United States; 3 Department of Public Health, Victoria University, Melbourne, VIC, Australia; 4 Department of Medicine, The University of Melbourne, Parkville, VIC, Australia; 5 Werribee Mercy Hospital, Werribee, VIC, Australia

**Keywords:** cardiovascular disease, chronic care model, dental care, general practice, heart disease, hypertension, integrated care, oral health

## Abstract

**Background:**

Poor oral health is associated with cardiovascular diseases (CVDs), largely due to shared risk factors such as smoking, diabetes and socioeconomic disadvantage. Integrating oral health into primary and specialist care, particularly cardiac services, presents a promising strategy to improve early detection of oral diseases and related health outcomes. This systematic review examines oral health strategies implemented in primary and cardiovascular care settings, focusing on their impact on oral and CVD indicators, service delivery and medical-dental collaboration.

**Methods:**

A systematic review was conducted in accordance with the Joanna Briggs Institute (JBI) Manual for Evidence Synthesis and reported following PRISMA 2020 guidelines, with the protocol registered in PROSPERO. Peer-reviewed studies were systematically searched across major databases. Guided by the PICO framework, data were extracted on populations, interventions, settings and outcomes. Study quality was assessed using JBI and Mixed Methods Appraisal Tool criteria, and findings were synthesised using narrative and thematic analysis.

**Results:**

Evidence from 17 studies highlight key integration strategies: (1) medical-dental collaboration through interdisciplinary care and co-location; (2) implementing oral health screening in non-dental settings; (3) non-dental teams providing oral health education and preventive care and (4) upskilling non-dental providers. Reported outcomes included improved oral health access, earlier detection of oral disease, enhanced provider confidence, increased chronic disease screening in dental settings and improved CVD markers.

**Conclusion:**

Integrated care models can bridge the oral-CVD care divide, improving equity and outcomes. However, sustainable implementation will require policy support, workforce training and evaluation of long-term cost-effectiveness and impact.

**Systematic review registration:**

https://www.crd.york.ac.uk/PROSPERO/view/CRD420251113478, PROSPERO CRD420251113478.

## 1 Introduction

In recent years, the traditional view of oral health has shifted, with growing evidence showing a bidirectional relationship between oral and systemic health (1). Evidence reports that oral diseases can lead to microbial imbalance (dysbiosis) and systemic inflammation, potentially contributing to chronic conditions such as COPD, Alzheimer’s disease, dementia, diabetes, stroke, and atherosclerotic cardiovascular disease (CVD) (2–6). Similarly, emerging evidence links Periodontitis, a chronic inflammatory condition caused by poor oral hygiene, with cardiovascular diseases – this is thought to occur through periodontal pathogens driving systemic inflammation and contributing to atherosclerosis (7, 8). Poor oral health has also been strongly associated with infective endocarditis in vulnerable cardiac populations and aspiration pneumonia in hospitalised stroke patients (9, 10).

Due to systemic consequences of poor oral health, there is growing consensus that integrating oral health care into general health systems can help maintain overall health and wellbeing due to the bi-directional relationship between oral health and non-communicable conditions (11). In November 2023, the FDI World Dental Federation, hosted a roundtable to highlight the vital role of oral health in overall health outcomes, emphasising that collaboration between oral health and primary care teams can position them as key advocates, enablers and mediators in advancing integrated healthcare (12). Such integration would not only target shared risk factors between oral and non-communicable diseases but will also support earlier detection of systemic conditions, improves data sharing, enhances health surveillance and enables more effective use of resources and workforce across the health system (11). However, oral health care continues to operate in relative isolation, with a predominant focus on treatment rather than prevention, posing a significant barrier to holistic care (11).

Internationally, dental care systems vary widely in structure and funding, but their limited coverage for adult dental services remains common and often neglected across the globe (13). In many countries, both high-income and developing, oral health is often given low priority and viewed more as an individual responsibility rather than a broader social one (14). Although few countries are progressing towards universal health coverage that includes heavily subsidising dental services for all population groups, but in many regions dental care is either excluded or only partially included in universal health insurance schemes, with preventive and routine services often funded to specific groups such as children and low-income populations (14).

In Australia, Medicare serves as the universal public health insurance system, providing free or subsidised medical care for citizens and eligible overseas visitors. However, dental care has largely been left out from Medicare, similar to many high-income countries like UK or New Zealand, and it only covers free dental for Australian children below the age of 18 under Child Dental Benefit Schedule (15). Due to its exclusion from Medicare, most dental care in Australia is delivered through private practitioners or private health insurance, resulting in substantial out-of-pocket costs for majority of consumers (16). Public dental services, including dental hospitals, function as a safety net for eligible/vulnerable populations and those requiring urgent care; however, due to fragmented funding, service capacity is limited and waitlists for routine treatment are painfully long (17). Due to these barriers, oral health remains inaccessible and a ‘luxury’ particularly for groups facing entrenched disadvantage (18).

Oral health is also not routinely prioritised in annual screenings and cardiovascular treatments and many individuals with heart conditions do not seek dental care, despite the emerging evidence emphasising its association (19). Furthermore, clinicians and nurses, who provide care to cardiac patients in their everyday routine setting, have further reported limited training and education on oral care, affecting how they address and practice oral health within their care (20).

Fragmented care between oral health and other health systems, such as cardiovascular care, may reflect limited evidence supporting cross-disciplinary collaboration. This gap has contributed to reduced oral health knowledge among clinicians and limited development of competencies needed to deliver coordinated, patient-centred care.

A systematic review was therefore conducted to map the available international evidence on oral health integration within these settings, and to identify opportunities for its application in the Australian context. It also aimed to evaluate and synthesise the impact of existing strategies, interventions and tools supporting integration in primary and specialised care on both oral and cardiovascular outcomes (e.g., oral hygiene, periodontal indices, blood pressure and inflammatory markers), as well as healthcare delivery outcomes, including referral pathways, patient experience and interdisciplinary collaboration.

## 2 Methods

A systematic review of oral health integration strategies for improved CVD and oral health outcomes was conducted in accordance with the Joanna Briggs Institute (JBI)‘s Manual for Evidence Synthesis (21). To enhance transparency in reporting, the Preferred Reporting Items for Systematic Reviews and Meta-Analyses (PRISMA) 2020 checklist were followed (22). The study selection process was documented using a PRISMA flowchart to clearly illustrate the screening and inclusion process.

The study protocol was registered in the international PROSPERO (International Prospective Register of Systematic Reviews) database (PROSPERO ID: CRD420251113478).

### 2.1 Selection criteria

In this systematic review, the PICOS framework (Population, Intervention, Comparator, Outcomes and Study Design) (23) was used to guide the development of eligibility criteria and inform the search strategy. PICOS framework provided a clear and structured approach to defining the review question, facilitating a systematic and transparent process for identifying and selecting relevant studies (Table 1).

**Table 1:** PICOS framework for eligibility criteria.

PICOS elements	Eligibility description
Population (P)	Individuals receiving or providing care within cardiovascular disease (CVD) services or primary healthcare settings.
Intervention (I)	Strategies integrating oral health into CVD or primary care (e.g., collaborative care models, training/upskilling non-dental staff, structured referral pathways).
Comparator (C)	Usual cardiac or primary care without integrated oral health components.
Outcomes (O)	Cardiovascular outcomes (e.g., changes in CVD events; inflammatory biomarkers; hospitalisation) Oral health outcomes (e.g., change in periodontal indices; oral hygiene status; oral hygiene behaviours/awareness) Patient-reported outcomes (e.g., quality of life, self-management) System-level outcomes (e.g., service uptake and/or improved cross-disciplinary collaboration)
Study design (S)	Experimental, analytical, mixed-methods, qualitative, service evaluations, published in English from 2015 onwards.

Studies were not restricted by country to capture a broad evidence base, with the intention of informing applicability to the Australian context. To ensure relevance to current policy landscape, only studies published in English from January 1, 2015, onwards were included. This reflects major shifts in the oral health considerations over the past decade, including Australia’s National Oral Health Plan (2015–2024) (24) and the United Nations Sustainable Development Goals (SDGs) (25). While oral health is not explicitly stated in the SDGs, it is closely linked to achieving them, particularly Goal 3 (Good Health and Wellbeing) which aimed at reducing communicable and non-communicable diseases by 2030 (25). This review therefore focuses on interventions aligned with these more recent policy directions.

Grey literature was excluded due to feasibility, as identifying and appraising such sources could introduce methodological challenges and may have compromise the transparency and reproducibility of the review.

### 2.2 Search strategy

A comprehensive search strategy was developed in collaboration with a research librarian and implemented across multiple electronic databases including EBSCOhost (covering Academic Search Ultimate, eBook Academic Collection, MEDLINE, CINAHL Ultimate, MEDLINE Ultimate and Australia/New Zealand Reference Centre Plus), Scopus and ProQuest Central. The search strategy combined both Medical Subject Headings (MeSH) and free-text keywords using Boolean operators and proximity searching. For search concepts and key terms see Table 2.

**Table 2:** Search terms used for systematic review.

Concept	Search terms
Oral Health	“oral health,” “mouth diseases,” “dental care,” “oral hygiene,” (oral OR gum OR periodontal) AND (health OR disease* OR hygiene)
Cardiovascular Disease	“cardiovascular diseases,” (cardio* OR cardiac OR heart) AND (disease* OR risk*), “hypertension,” “myocardial infarction,” “stroke,” ‘heart failure’,
Integrated Care	“integrated care,” “chronic care model,” “intersectoral collaboration,” “delivery of health care, integrated,” “patient care team,” “team-based care”
Primary Care	“primary health care,” “general practice,” “community health services,” “primary care nursing”

Search terms were adapted for each database interface. Proximity operators (N2, N3) were used where supported to capture terms appearing within two or three words of each other.

In addition to comprehensive database searches, targeted hand searching was conducted on google scholar and other journals and sources known to publish research on integrated oral and cardiovascular care. This approach ensured the inclusion of potentially relevant studies that may not have been captured through electronic database indexing.

### 2.3 Article selection process

**Figure 1:**
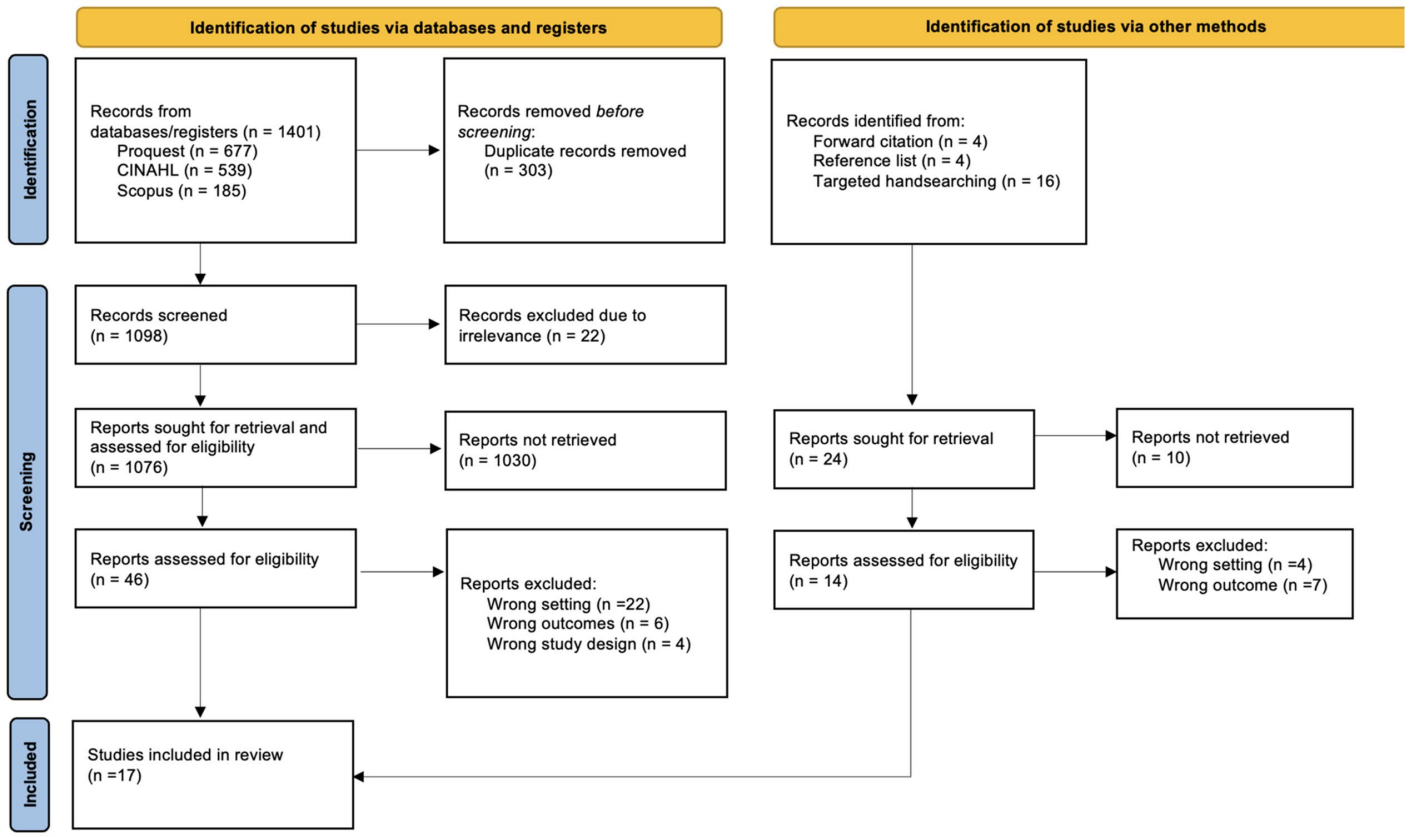
PRISMA flow chart (22).

All records identified through the search were imported into Covidence for management and screening. Two independent reviewers (SO, MR) screened all titles and abstracts against the inclusion criteria. Full-text articles were retrieved for studies deemed potentially eligible or where eligibility was unclear from the abstract. Two independent reviewers (WU, CP) then assessed the full-text articles for final inclusion and conducted data extraction. Any disagreements during the screening or eligibility assessment stages were resolved through discussion and when necessary, a third reviewer (FH) was consulted. The study selection process, including reasons for exclusions at the full-text stage, was documented using the PRISMA flow diagram (22) (see Figure 1).

### 2.4 Data extraction and synthesis

Data extraction was conducted using a standardised template within Covidence, a systematic review management platform. Key study characteristics such as author, year, country and study design, as well as details on sample population was retrieved (see Table 3). Information related to how integration models of oral health into cardiovascular or primary care such as the roles of health professionals, oral care delivery methods and broader contextual or system-level features and their policy implication were also recorded (see Table 4). Any differences in extracted data were discussed and resolved by consensus, with a third reviewer consulted in case of data extraction conflict. Authors of the included studies were contacted for clarification when data were missing or unclear.

**Table 3:** Study characteristics.

Study ID	Study location	Study design	Study sample and size	Study setting	Study quality
Ab Malik et al. (44)	Malaysia	Randomised control trial	547 stroke care providers across 10 hospitals	Rehabilitation/stroke unit (hospital) setting	High
Cardenas et al. (40)	USA and Somaliland Africa	Mixed-method	17 primary care and dental clinics	Primary care and dental clinic setting	High
Dai et al. (35)	Hong Kong	Randomised control trial	94 patients with stroke	Outpatient rehabilitation setting	High
Eto et al. (34)	Japan	Retrospective cohort	419 acute ischaemic stroke patients	Rehabilitation/stroke unit hospital setting	Moderate
Hashem et al. (28)	UK	Service evaluation	Service evaluation of 851 dental referrals from the dental care pathway (study sample /size is not applicable)	Hospital setting	Moderate
Huang et al. (36)	Taiwan	Quasi experimental	136 community adults with cardiometabolic syndrome, living in rural areas of Taiwan.	Not specified	High
Kim et al. (37)	Korea	Retrospective cohort	478,245 individuals aged 40–79 years	Primary care and hospital setting	High
Kohli et al. (39)	USA	Cross-sectional	20 registered nurses mostly females actively licensed and with geriatric experience.	Nursing school setting	High
Lim et al. (38)	Korea	Longitudinal cohort	Data from 5,413 participants in the Korean Longitudinal Study of Aging.	Not specified	High
Mihart et al. (42)	Romania	Case control	84 participants (40 males and 44 females) with ages between 50 and 89 years old, hospitalised in the cardiology unit	Hospital inpatient setting	High
Mourtison et al. (29)	USA	Service evaluation	Healthcare providers and dental patients who received care at or a part of six federally qualified health centres in Colorado that implemented the Diabetes Cardiovascular Disease Oral Health Integration program (DCVDoHI)	Primary care and dental clinic setting	Low
O’Rorke et al. (27)	UK	Service evaluation	Children aged 288 children aged 0-18 years with congenital heart disease (CHD)	Cardiac outpatient setting	High
Obana et al. (33)	Japan	Cross-sectional	115 stroke patients who were admitted to a university hospital in Tokyo	Hospital inpatient setting	High
Omori et al. (32)	Japan	Quasi experimental	70 hospitalised patients with heart disease	Hospital inpatient setting	High
Sanchez et al. (43)	Australia	Cross sectional	321 participants with and without heart disease	Cardiac rehabilitation and cardiology specialist setting	High
Schulz et al. (45)	Germany	Quasi experimental	107 children with congenital heart disease (CHD) aged between 2 and 6 years	Primary, cardiac specialist and dental clinic setting	High
Smith et al. (41)	UK	Mixed-methods	Inpatients with confirmed stroke who required assistance with personal care and stroke unit nursing staff who received oral hygiene education and training. Sample size is unclear.	Rehabilitation/stroke unit (hospital) setting	Moderate

**Table 4:** Summary table of oral health integration strategies.

Study ID	Integration strategies	Description
Cardenas et al. (40)	Medical and dental collaboration.	A learning collaborative (TISH) was developed which included biweekly virtual sessions with primary and oral health teams on integrating care to improve hypertension screening in dental settings and gingivitis detection in primary care and increase bidirectional referrals.
Mauritson et al. (29)	Policy change	The integration program (DCVDOHI) provided grants to six federally qualified health centers (FQHCs) within Colorado which typically offer comprehensive primary care services, including medical and often dental, to underserved populations. These grants aimed at improving medical and dental collaboration.
	Medical and dental collaboration	The program promoted closed-loop referrals, co-located dental hygienists in medical practices to detect oral diseases, conversely dental patients were also screened for chronic diseases.
Hashem et al. (28)	Medical and dental collaboration	Inpatients were referred via a hospital dental care pathway, triaged and assessed within 1 day for urgent treatment or surgical referral. Only admission-related issues were treated; routine problems deferred. The pathway aimed to improve oral health access for admitted patients.
Obana et al. (33)	Medical and dental collaboration	Transdisciplinary care was provided to stroke patients where stroke nurses used oral health screening tool for initial and weekly assessments with daily care; dentists and speech therapists provided weekly treatment based on needs; weekly multidisciplinary team (stroke nurses, dentists, speech therapists) meetings were held using a shared oral health sheet to support collaboration.
Kim et al. (37)	Oral health screening in non-dental setting	In South Korea, adults receive free biennial health screening for CVD prevention, including checks for hypertension, diabetes, dyslipidaemia and a separate oral health screening. The oral health screening protocol (OHSP) in this program involves questionnaires, examinations and counselling, with recommendations for further dental care if needed.
Lim et al. (38)	Oral health screening in non-dental setting	Geriatric Oral Health Assessment Index (GOHAI) was used to evaluate oral health related quality of life in patients diagnosed with CVD (including cerebrovascular disease and transient ischemic attack).
Mihart et al. (42)	Oral health screening in non-dental setting	The total number of remaining permanent teeth (NRT) on both arches was assessed in cardiac patients from the cardiology department to evaluate its potential as a predictive risk factor for a specific cardiovascular condition.
Eto et al. (34)	Oral health screening in non-dental setting	Oral health assessment was conducted using modified oral assessment grade (mOAG) among hospital in patients with acute stroke. Oral care was provided to the participants based on the mOAG scores. For patients with lower mOAG scores and those without consciousness impairments, oral hygiene guidance was provided to educate and empower them to maintain their oral health.
Sanchez et al. (43)	Oral health screening in non-dental setting	A four-item screening tool, COH, was developed by an expert panel and validated through a cross-sectional survey of patients with cardiovascular disease in cardiac care settings. This tool was developed to identify patients at poor risk of oral health.
O’Rorke et al. (27)	Oral health screening in non-dental	A paediatric dental screening service for children with CHD was delivered by a dental therapist within cardiology clinics at London; screenings used a proforma, preventive advice was given and tailored reports were sent to dentists, cardiologists and families.
Hunag et al. (36)	Oral health education/preventive care	An oral hygiene program was developed for rural adults with metabolic syndrome, combining outpatient oral health education with follow-up health-promoting counselling via phone.
Dai et al. (35)	Oral health education/preventive care	Advanced Oral Hygiene Care Programme (AOHCP) was developed to improve oral hygiene and reduce gingival bleeding in stroke patients, participants received a powered toothbrush, chlorhexidine mouth rinse, toothpaste and oral hygiene training in an outpatient rehabilitation clinic.
Schulz-Weidnar et al. (45)	Oral health education/preventive care	Children with congenital heart disease participated in a preventive oral health program based on an established regimen in preschools in Germany. This program included oral health education by the attending dentist during cardiology appointments with follow-up measurements of oral hygiene and gingival bleeding, reinforced by additional training on oral hygiene and re-motivation at subsequent cardiology recall visits.
Omori et al. (32)	Oral health education/preventive care	Oral preventive care program including was administered to hospital in patients with atrial fibrillation. This program included preoperative mechanical tooth cleaning, scaling and tongue coating removal in a single dental visit, followed by intensive, theory-based oral hygiene instruction using a six-step self-efficacy method with barrier identification, goal-setting, self-monitoring and relapse prevention.
Kohli et al. (39)	Training/education for non-dental providers	Nurses with geriatric expertise received online training called” Smiles for Life,” which included five modules and supervised clinical practice using the Oral Health Screening Tool for Nursing Practice (OHSTNP).
Smith et al. (41)	Training/education for non-dental providers	As part of the oral health preventive intervention for stroke patients, online training in relation to oral health education and management was provided to the stroke nursing staff with practical sessions. Oral health protocol was then implemented among stroke inpatients that consisted of brushing the teeth twice a day with a chlorhexidine gel or a non-foaming toothpaste with denture care when necessary.
Ab Malik et al. (44)	Training/education for non-dental providers	A web-based continuing professional development (CPD) program was provided to stroke care providers. It was based on oral hygiene care and its provision to stroke patients and covered details of oral health knowledge, attitudes, subjective norms, means of behavioural control and intention (i.e., based on theory of planned behaviour).

To synthesise findings across diverse study types, a thematic analysis was undertaken across all included studies. This followed Braun and Clarke’s six-step approach (26) which included getting familiar with the data, coding relevant content, developing and refining potential themes and finally defining and presenting themes that captured patterns on how oral health care had been integrated in non-dental settings. This method allowed for the identification of common strategies, approaches and contextual factors that supported or hindered integration across settings.

### 2.5 Quality appraisal

The methodological quality of included studies was assessed using critical appraisal tools appropriate to each study design. The Joanna Briggs Institute (JBI) tools were applied to cross-sectional studies case control studies cohort studies and randomised controlled trials (RCTs) (21).

For service evaluation studies (27–29) JBI case series checklist was used, as no dedicated tool for appraising service evaluations have been established in literature. While these studies were not strictly case series in design, they did involve some level of observational data collection and descriptive analysis and due to this, using this appraisal tool was deemed the most appropriate. However, it may be a limitation, as these checklists may not fully capture the contextual aspects of service evaluations, potentially biasing their quality assessments.

This review adopted the scoring method used by Shu et al. (30) where the authors calculated quality scores from JBI appraisal tools by dividing the number of “Yes” responses by the total checklist items, then multiplying by 100 to yield a score percentage. Studies were then classified as low if they scored <50%, moderate if they score between 50 and 70% or high quality if they score >70%.

Mixed methods studies were assessed using the Mixed Methods Appraisal Tool (MMAT) (31). While the MMAT developers discourage numerical scoring in favour of descriptive, criterion-based appraisal, the authors did apply the same overall scoring method used for JBI tools to maintain consistency in how study quality was reported across this review.

Given the variation in methodological designs, each appraisal tool focused on relevant aspects for that design such as randomisation and blinding for RCTs, sampling methods and measurement reliability for cross-sectional studies and integration of qualitative and quantitative components for mixed methods studies. Quality scores were interpreted within the context of each study type and were not directly compared across designs. For example, a high-quality rating for a cross-sectional study reflects strong design and reporting within that format, but does not suggest greater evidentiary strength than a moderate quality RCT. This approach maintained methodological rigour while respecting the hierarchy of evidence during synthesis.

## 3 Results

A total of 1,422 records were identified through the search process. This included 1,401 records from database searches; ProQuest (*n =* 677), CINAHL (*n =* 539) and Scopus (*n =* 185) and 24 additional records identified through other sources, including forward citation searching (*n =* 4), reference list screening (*n =* 4) and targeted handsearching (*n =* 16). After removing 303 duplicates, 1,119 records (inclusive of records from other sources) remained for screening. In the first phase, titles and abstracts were assessed for relevance. The remaining articles underwent full-text review, those studies which did not meet the PICOS criteria were excluded. This extraction was conducted among 17 studies that were included in the final synthesis (Figure 1).

### 3.1 Study characteristics

The included studies covered a wide range of global settings, including Asia (32–38), North America (29, 39, 40), Europe (27, 28, 41, 42) and Australia (43), highlighting the diversity of contexts in this review. Study designs varied considerably. Two studies used randomised controlled trials (35, 44), while others adopted quasi-experimental (32, 36, 45), cohort (34, 37, 38), case–control (42), and cross-sectional designs (33, 39, 43). Mixed-methods studies (40, 41) and service evaluations (27–29) were also included. These studies involved participants with or at risk of cardiovascular disease, as well as primary care and hospital-based clinicians.

Study settings spanned across primary, secondary, and specialised care, including rehabilitation and cardiology clinics (as shown in Table 4).

### 3.2 Risk of bias

Thirteen studies were appraised as high quality (27, 32, 33, 35–40, 42–45). These studies were characterised by clear inclusion criteria, rigorous methodology and comprehensive reporting. Despite their overall strength, minor limitations were identified. For example, Obana et al. (33) excluded participants due to missing data without specifying affected variables, reducing transparency. Kohli et al. (39) collected demographic information relevant to oral health knowledge but did not adjust for these factors, partly due to a small sample size. Although Sanchez et al. (43) evaluated a validated screening tool, partial reliance on self-reported measures may have introduced response bias. While the included RCTs and cohort studies were generally high quality, some were limited by incomplete blinding and short follow-up periods (35, 37, 38, 44). O’Rorke et al. (27) service evaluation, acknowledged that non-validated clinical screening methods may have underestimated dental disease prevalence. Cardenas et al. (40) demonstrated strong mixed-methods integration, though the quantitative component was limited by sample representativeness and potential nonresponse bias.

Three studies (28, 34, 41) were appraised as moderate quality with a moderate risk of bias. Hashem et al. (28), reported referral patterns but lacked standardised diagnostic criteria, did not mention the characteristics of the participating adults and limiting consistency. Eto et al. (34) was limited by incomplete follow-up and lack of external validation of the screening tool. Smith et al. (41), a mixed-methods feasibility study, demonstrated strong qualitative integration but the quantitative arm was weak due to limited representativeness, absence of confounder acknowledgement/adjustment and incomplete documentation of protocol deviations. These limitations, typical of feasibility studies, constrained the generalisability of the results.

Mauritson et al. (29) was appraised as low quality as it did not state inclusion and exclusion criteria for participating clinics, there was no indication of using standardised tools for measuring exposure and outcomes. Since this study was conducted as a service evaluation it only had limited elements of a descriptive statistics and therefore did not meet the methodological standards typically expected of observational and descriptive research. This study was included for contextual insight, however, its findings are valuable in understanding the impact of inter-disciplinary care.

### 3.3 Thematic analysis of the included studies

The findings of this review are organised into four key thematic strategies: (1) patient-focused oral health interventions; (2) medical-dental collaboration; (3) oral health screening in non-dental settings; and (4) workforce training. Table 4 below summarises the types of integration implemented in the identified studies and the outcomes measured to evaluate their impact.

#### 3.3.1 Interprofessional (medical-dental) collaboration

Four studies focused on interprofessional collaboration (28, 29, 33, 40). As illustrated in Table 5, several interdisciplinary initiatives demonstrated measurable service-level outcomes. For instance, the Teaming and Integrating for Smiles and Health (TISH) learning collaborative (40) established bidirectional referral pathways between dental and primary care. Across participating dental sites, hypertension screening identified substantial unmet need, with one site screening 861 patients and referring 83 (9.6%) to primary care, a second screening 409 patients and referring 59 (13.3%), and a third referring 557 patients (14.7%). Evaluation data indicated improvements in screening volume, referral activity, and interprofessional workflows, although gingivitis screening and referral activity in primary care remained minimal and did not reach statistical significance (40).

**Table 5:** Summarised results of medical and dental collaboration strategies.

Study ID	Method of collaboration	Outcomes measured	Findings
Cardenas et al. (40)	Medical and dental referral pathways; virtual seminars for medical and dental teams on integration	Screening rates for hypertension and gingivitis; clinician knowledge and attitudes related to oral health.	Improved hypertension screening and referrals to primary care in dental settings. Little to no change in gingivitis screening/referral in primary care. Improved medical workflows and medical–dental communication. Improved awareness of oral–primary care links among staff and patients.
Obana et al. (33)	Interdisciplinary communication and meetings; oral health assessments; collaborative care plans	Oral health assessment tool (OHAT) scores on admission and discharge	Interdisciplinary care significantly reduce stroke patient’s OHAT scores, meaning imporved oral health status was observed on patient’s discharge.
Mauriston et al. (29)	Medical and dental co-location; medical and dental referral pathways.	Prevalence, control and screening of hypertension and diabetes; referrals for medical and dental care; ED visits.	Increased screening of dental patients for chronic diseases, including hypertension across participating clinics. Reduced ED visits among patients who had two or more diagnostic dental visits.
Hashem et al. (28)	Medical and dental co-location; medical and dental in-patient referral pathways	Referral outcomes (reason for referral/referral rates)	The most common reason for referral was dental pain sepsis or infection and assessment of oral cavity prior to cardiac surgery. Many referrals resulted in basic management (advice or extraction), showing limited scope of interventions provided. Increase in overall inpatient-to-dental referrals since 2014.

Similarly, the Colorado Diabetes Cardiovascular Disease Oral Health Integration Program (DCVDOHI) (29) reported large-scale screening outcomes across six medical–dental teams. Between 2019 and 2020, over 9,500 dental patients were screened, with 60% assessed for hypertension and 16% for diabetes risk. Among those screened, 26% had elevated blood pressure and 34% of point-of-care HbA1c tests indicated possible diabetes. Medicaid claims analysis demonstrated that patients receiving multiple dental visits or periodontal treatment had approximately a 60% lower hazard of emergency department visits compared with those receiving fewer or no dental services.

Clinical improvements were also observed in hospital-based transdisciplinary models. Obana et al. (36) reported significant improvements in oral health among acute stroke patients receiving coordinated care involving dentists and dental hygienists (OHAT assessments and bedside professional care), nurses (basic oral care) and speech therapists. Care was supported by weekly multidisciplinary meetings and shared oral health documentation. Median OHAT scores improved from 4 at admission to 3 at discharge, with gains across most domains except dentures and dental pain; poorer discharge oral health was associated with higher baseline scores for tongue condition, dentures and oral cleanliness.

Co-located medical–dental services further supported early detection and management of oral disease. In a service evaluation of a UK hospital dental care pathway, Hashem et al. (28) analysed 851 inpatient referrals to hospital’s maxillo-facial department were made. Referral activity increased by 34% compared with 2014 levels, and most referrals were triaged within one working day. Common outcomes form this referral pathway included consultation or advice (16%) and dental extraction (16%).

#### 3.3.2 Oral health screening interventions in non-dental settings

Six studies evaluating health screening delivered outside traditional dental settings, predominantly in cardiovascular and stroke care (27, 34, 37, 38, 42, 43), demonstrated its utility for early risk identification and prediction of adverse outcomes (Table 6).

**Table 6:** Summarised results of oral health screening interventions.

Study ID	Oral health screening tools/methods	Outcomes measured	Findings
O’Rorke et al. (27)	Visual examination (using disposable mirror and troch) and screening proforma developed by research team.	Access to dental care; oral hygiene practice; oral hygiene status; gingival inflammation; dental caries; dental infection; urgent referral needs.	The early oral health screening proforma identified: 31% of the population (children with CHD) were not registered with a local dentist and did not have regular dental access. 46% reported adequate teeth brushing habits. Clinical examination showed that 19% had poor oral hygiene. 14% had visible gingival inflammation. 16% had visible dental caries and 1% had active dental infections with associated intraoral signs. 11% required urgent referral to a paediatric dental centre for specialist dental treatment.
Kim et al. (37)	General dental screening included as a component of a broader Korean national health screening program.	Major adverse cardiovascular events (MACE); all-cause deaths; healthcare/hospital utilisation	The risk of major adverse cardiovascular events was 10% lower in general health and dental screening group when compared with non-screening group and 9% lower than those who only went for general screening. The dental screening group was associated with low all-cause deaths. Healthcare utilisation was lower in the dental screening group but was not statistically significant.
Lim et al. (38)	Geriatric Oral Health assessment index (GOHAI)	CVD diagnosis; oral health related quality of life (OHRQoL)	Poor GOHAI scores (<40) were significantly associated with increased risk of CVDs, even after various confounding factors. Stronger association observed in females than males
Mihart et al. (42)	Oral parameter reflecting the total number of remaining permanent teeth (NRT)	Number of remaining teeth; presence of CVD conditions; systematic comorbidities	Tooth loss (<21 remaining teeth) was a strong predictor of cardiomyopathy, heart valve disease and hypertension, especially once other factors (like age, systematic comorbidities, lifestyle) were taken into account. No significant link was found with coronary heart disease or arrhythmias. The strongest association by far was with cardiomyopathy, where people with fewer teeth were tens of times more likely to have the condition.
Eto et al. (34)	Modified oral health assessment grade (mOAG)	Functional stroke outcome; hospital acquired pneumonia (HAP)	The mOAG scores were found to be effective in predicting stroke outcomes: Lower mOAG score at admission was independently associated with good stroke outcomes and vice versa. Lower mOAG scores at admission were also independently associated with a decreased incidence of HAP.
Sanchez et al. (43)	Cardiovascular Oral Health (COH) screening tool	Frequency of dental visits; periodontal disease; smoking status; xerostomia (dry mouth)	COH tool is highly sensitive (88–89%), effectively identifying patients with poor oral health. It has low specificity (24–33%) means it may flag patients as at risk even if oral health is fine, potentially causing over-referrals. Significant correlations with OHIP-14 (gold standard) and clinical exams (gold standard) confirm the tool’s validity. Prioritises catching all at-risk patients over precision, which is appropriate in CVD populations to prevent serious complications.

A population-based cohort study from South Korea (37) reported that participants who had undergone dental screening demonstrated a significantly lower risk of major adverse cardiovascular events (MACE) compared to no screening (adjusted HR 0.90; 95% CI 0.87–0.93) and general health screening alone (adjusted HR 0.91; 95% CI 0.89–0.94). All-cause mortality was also lower in the dental screening group, alongside reduced CVD-related healthcare utilisation, although healthcare costs did not differ significantly.

Screening tools assessing oral health status were examined in stroke and older adult populations to predict the risk of hospital-acquired pneumonia (34). In acute ischaemic stroke, higher modified Oral Assessment Grade (mOAG) scores at admission were associated with poorer functional recovery and increased risk of hospital-acquired pneumonia, with each one-point increase in mOAG increasing the odds of poor outcome (OR 1.31) and pneumonia (OR 1.21) (38). Another study, using data from 5,413 older adults in the Korean Longitudinal Study of Ageing (2018–2020) (38) examined the link between oral health related quality of life and CVDs, using the Geriatric Oral Health Assessment Index (GOHAI). Poor GOHAI was significantly associated with increased odds of CVD (OR 1.13, 95% CI 1.07–1.19), even after adjusting for demographic and clinical risk factors, with a stronger association observed in females (OR 1.36) than males (OR 1.12). The prevalence of poor GOHAI increased from 51.2 to 70.3% between 2018 and 2020, alongside a rise in CVD prevalence (15.2–17.0%), likely reflecting the cohort’s ageing profile (38).

Rapid screening tools were also evaluated to predict the risk of developing CVDs. For instance, a Romanian hospital-based case–control study reported that number of remaining teeth (NRT) could be a predictor of certain CVD conditions. The study found that fewer than 21 remaining teeth was strongly associated with cardiomyopathy, heart valve disease and hypertension after adjusting demographic factors such as age, gender and residence (OR = 15.56) and further adjusting for systematic conditions such as pulmonary disorders, metabolic disorders, chronic renal failure (OR = 78.23) (42). While, in an Australian hospital setting, a four-item rapid Cardiovascular Oral Health (COH) screening tool demonstrated high sensitivity (≥88%) but low specificity (<35%) when validated against clinical examination and gold standard tools such as Oral Health Impact Profile (OHIP-14) (43).

Dental screening tool in paediatric cardiology cohort was also studied (27). Among children with congenital heart disease, dental screening proforma detected increased proportion of ument dental needs: poor oral hygiene, gingival inflammation, visible caries and dental pain, with over one in 10 requiring urgent specialist referral.

#### 3.3.3 Oral health education/interventions in non-dental settings (CP)

Four studies (32, 35, 36, 45) investigated the impact of oral health education on cardiovascular health in non-dental settings (see Table 7).

**Table 7:** Summarised results of oral health preventive care/ interventions.

Study ID	Oral health interventions	Outcomes measured	Findings	Follow-up period
Hunag et al. (38)	Free oral hygiene products, group and individual oral health education, home-phone counselling	BMI, body fat; triglyceride; tooth scaling incidence	Intervention group showed greater decrease in BMI, body fat (*p <* 0.001), triglyceride levels (*p <* 0.05) Neither group showed significant change in blood pressure (*p* > 0.9) Oral hygiene behaviour in the intervention group improved more significantly than the control group (*p <* 0.001 for brushing teeth, using dental floss, oral discomfort, tooth scaling regularly)	12 months
Dai et al. (35)	Oral hygiene instruction/training (advanced vs. conventional), free oral hygiene products	Gingival bleeding index; plaque index	Participants who received advanced oral hygiene instruction and used chlorhexidine mouth wash had higher teeth staining and tartar levels than the conventional group (*p <* 0.05) Participants who received advanced oral hygiene instruction had a smaller percentage of gingival bleeding sites than the conventional group (*p <* 0.01) Tooth brushing was more effective at reducing plaque levels than gingival bleeding	3-6 months
Schulz-Weidner et al. (45)	Preventive oral hygiene program in preschools, dental check-up	Gingival index; plaque index; dental caries signs; tooth brushing technique	Children with congenital heart disease had significantly higher gingival indices, plaque values and increased treatment needs than controls After intervention, GI of CHD group decreased significantly by 38.46% (*p <* 0.01) CHD group also saw a 16.6% decrease in mean plaque values after intervention (*p <* 0.001)	6-12 months
Omori et al. (32)	Six-step behavioural oral health education	Tongue bacteria count, plaque levels, postoperative atrial fibrillation, gum health	Oral health behaviour (frequency of tooth brushing and interdental brushing) improved in the intervention group than the control (94.3% vs. 54.3%) The intervention group had less oral bacteria on the tongue after intervention compared to the control group (*p <* 0.001) Postoperative atrial fibrillation period was shorter in intervention group compared to control (*p* = 0.019)	2 weeks

Huang et al. (36) experimental study sorted 160 participants with metabolic syndrome in rural Taiwan into intervention and control groups. Both groups received toothbrushes, floss, group oral health education and private education on healthy habits like physical activity, health eating and stress management. The intervention group also received a pedometer to record the number of steps/daily exercise and monthly home-phone counselling 2 weeks after receiving the oral hygiene materials/education. The 5–10-min counselling sessions served to remind participants of oral hygiene habits and discuss healthy lifestyle. After the 12-month follow-up period, the intervention group showed greater decrease in BMI (*p <* 0.01), body fat percentage (*p <* 0.05) and triglyceride levels (*p <* 0.05) than the control group. The intervention group also had greater use of dental floss (*p <* 0.05), regular tooth scaling (*p <* 0.05), physical activity (*p <* 0.001) and health promoting behaviours (*p <* 0.001) compared to the control group (36).

Dai et al. (35) and Omori et al. (32) compared advanced/supplemental oral hygiene programs to standard versions. Dai’s randomised controlled trial in Hong Kong included 94 stroke rehabilitation patients who received either an advanced or conventional oral hygiene care program over 6 months. The advanced program incorporated a powered toothbrush and chlorhexidine mouth rinse in addition to standard oral hygiene training and toothpaste. Both groups demonstrated significant reductions in gingival bleeding, while the advanced program group showed a significantly greater reduction in moderate to abundant plaque at 3 months (*p <* 0.01). Omori’s quasi-experimental study in Japan evaluated an enhanced oral health instruction program among 70 cardiac surgery patients who all received mechanical tooth cleaning. Participants in the intervention group additionally received weekly 15-min oral hygiene instruction based on a six-step, self-efficacy–focused approach. After 2 weeks, the intervention group showed significantly greater reductions in tongue coating, oral bacterial counts and plaque scores compared with controls (*p <* 0.001) (32) (Tables 8, 9).

**Table 8:** Summarised results of workforce and training interventions.

Study ID	Models of oral health training	Outcomes measured	Findings	Follow-up period
Kohli et al. (47)	Interprofessional education (online, self-paced didactic training program followed by hands-on clinical training)	Nurses’ knowledge improvement; nurses’ confidence in providing oral screenings and patient satisfaction	Training effectively boosted nurses’ oral health knowledge and confidence. High patient satisfaction was also observed regarding oral screening provided by nurses’ post training.	Immediate post training evaluation was conducted (period not specified)
Smith et al. (44)	Protocol-based clinical training model through a combination of online learning and practical, hands-on sessions.	Feasibility of intervention; adherence to intervention; staff knowledge confidence; program’s safety outcomes	Nurses reported greater confidence and competence in providing oral hygiene care and became more consistent in performing mouth cleaning and denture care. Improved patient comfort, communication and overall wellbeing, suggesting that regular oral care contributed to better rehabilitation outcomes	N/A
Ab malik et al. (37)	Web based continuing professional development model that was structured around the Theory of Planned Behaviour (TPB).	TPB components (attitude; subjective norm; perceived behavioural control; general intention; knowledge)	TPB-based CPD model effectively changed participants’ attitudes, social influences and intentions toward providing oral hygiene care. No significant change observed in perceived behavioural control domain among test group.	Total follow-up period was 6 months, with data collected at baseline, 1 month and 6 months after the training.

**Table 9:** Outcome matrix.

Integration strategy	Oral health outcomes	Cardiovascular outcomes	Access/service delivery	Provider knowledge/skills
Oral health education and preventive interventions	✓	✓	✓	✓
Medical–dental collaboration	✓	–	✓	–
Screening in non-dental settings	✓	✓	✓	–
Workforce training	–	–	✓	✓

✓ = Improvement reported in included studies. – = Limited or no evidence reported.

Schulz et al. (45) was the only paediatric study that employed preventive oral hygiene education. Conducted in Germany, the study compared the oral health of 107 preschool-aged children with congenital heart disease and 101 healthy children. At baseline, children with congenital heart disease (CHD) had higher rates of dental caries, plaque values and signs of gingivitis and hyperplasia compared to healthy children (*p <* 0.05). Both groups participated in a preventive oral hygiene education program based on an established regimen in German preschools, including oral hygiene demonstrations, dental check-ups and encouraging tooth brushing and healthy nutrition. Children with CHD also began an intensive care program with a dentist. After 12 months, the gingival index of children with CHD decreased significantly (*p <* 0.01) and a decrease in mean plaque values (*p <* 0.001) (45).

#### 3.3.4 Building skills and knowledge in non-dental professionals

Three studies (39, 41, 44) examined how different forms of oral health training improved the knowledge, confidence and skills of nurses and other frontline health professionals to better address oral health needs and promote cardiovascular health outcomes.

Study by Kohli et al. (39) explored how interprofessional education (IPE) for non-dental professionals can improve their knowledge and confidence in providing preventive oral care and screenings. This study evaluated Smiles for Life program, endorsed by the American Dental Association and the American Academy of Family Physicians, combined online modules with supervised clinical practice to promote integration of oral health in primary care. Modules covered oro-systemic health, geriatric oral health, acute dental problems and infection control (39). Nurses first practised screening each other before assessing patients under the supervision of dental faculty and their performance was measured using the validated tool. Results showed significant improvements in oral health knowledge (mean scores increased from 38.33 to 47.93, *p <* 0.05) and confidence in discussing oral issues and referrals (from 35.7 to 64.3%), along with high patient satisfaction (97.1%).

Ab Malik’s randomised controlled trial evaluated a web-based continuing professional development program based on the Theory of Planned Behaviour among stroke care providers across 10 Malaysian hospitals. At one-month post-intervention, the test group demonstrated significant improvements in attitude (*p* = 0.004) and perceived social support from colleagues (*p* = 0.01). By 6 months, significant improvements were observed in intention (*p* = 0.003), attitude (*p* = 0.009), social norms (*p <* 0.001) and oral health knowledge (*p* = 0.001), while no significant change was detected in perceived behavioural control (44).

Smith et al. (41) conducted a feasibility study in a UK stroke unit assessing the implementation of a structured oral-care protocol delivery and its training delivered in stroke nurses. Following 6 weeks of online and practical training, the intervention was deemed feasible, with high acceptance, a 95% adherence rate to the protocol, and significant improvements in staff oral-care knowledge.

### 3.4 Synthesis of evidence

Across the 17 included studies, integration strategies consistently improved oral health outcomes and access to care, but evidence for cardiovascular benefits was limited. When weighting findings by study quality, the strongest evidence supports oral health education and preventive interventions, which were evaluated in multiple high-quality randomised controlled trials and quasi-experimental studies (32, 35, 36). These interventions demonstrated significant reductions in plaque scores, gingival bleeding, oral hygiene behaviours, providing high confidence in their effectiveness for oral health improvement.

Medical-dental collaboration models also showed positive effects on oral health indicators, oral health access and continuity of care, particularly in service evaluations and mixed-methods studies rated as high quality, some improvements were also seen in hypertension screening rates, in dental settings. Screening interventions in non-dental settings yielded promising results, with only one large cohort study linking dental screening to reduced major adverse cardiovascular events (37). Couple of oral care interventions also reported therapeutic reductions in post-atrial fibrillation period (32) and greater decrease in BMI, body fat and triglyceride levels (36). Yet, these findings lacked long-term follow-up or standardised outcome measures, limiting confidence in their impact on cardiovascular endpoints suggesting low confidence in cardiovascular benefits and highlighting the need for further research.

Workforce training interventions consistently improved provider knowledge and confidence, as reported in systematic reviews and cross-sectional studies of high methodological quality. However, few studies measured downstream patient outcomes, indicating that while training is an essential enabler, its direct effect on oral or cardiovascular health remains uncertain.

Overall, the synthesis indicates:

- High confidence for oral health improvements through screening, education and preventive care.
- High confidence for improved access and service delivery via collaboration and screening.
- Moderate confidence for improved non-dental provider skills/knowledge in relation to oral health and oral health care.
- Low confidence for cardiovascular outcomes due to limited high-quality evidence.

## 4 Discussion

This review synthesised evidence from 17 studies across diverse clinical and geographic contexts and identified four core integration strategies that consistently supported the inclusion of oral health within primary and specialised cardiovascular care pathways: (1) patient-facing oral health education and preventive interventions; (2) medical–dental collaboration through shared referral pathways and co-location; (3) oral health screening conducted in non-dental settings; and (4) workforce training for non-dental professionals. Across these strategies, studies reported improved access to oral health services, higher screening uptake, enhanced provider knowledge/confidence and measurable improvements in oral health indicators (e.g., plaque control, gingival bleeding) in stroke and cardiac populations (32, 35, 37). However, effects on cardiovascular biomarkers (e.g., blood pressure, lipids) were limited and typically modest or non-significant in short-duration interventions (36).

The included studies reported consistent limitations related to implementation, methodology and generalisability. Interventions were often constrained by time pressures, workforce shortages, limited resources, staff turnover and system-level barriers such as electronic health record incompatibility and testing costs (29, 41). Methodological limitations included reliance on self-reported oral health measures, missing or absent baseline oral health data, lack of comparison with gold-standard assessment tools and short follow-up periods, which limited causal inference and interpretation of outcomes (34, 36, 38). One study also acknowledged potential selection bias, residual confounding and difficulty isolating the independent effects of oral health screening from broader health behaviours, particularly when examining cardiovascular outcomes (37).

Generalisability was further restricted by small sample sizes, homogeneous or single-site populations and, in paediatric studies, absence of post-intervention assessment in healthy comparison groups (42, 45). Finally, screening tools were noted to carry a risk of over-referral and false positives, highlighting the need for further validation in larger and more diverse cardiovascular populations (43).

Moreover, among included reviews Implementation success varied by context, for instance, US Medicaid-supported initiatives demonstrated that co-location, closed-loop referrals, point-of-care testing and patient navigation, supported by system-wide funding can operationalise integration and reduce downstream utilisation (29). These findings argue for policy levers (funding, coding, digital interoperability) in combination with workforce development as well as incentivising non-dental professionals including primary care workers for providing basic oral care and education to further scale integrated care especially in rural and remote areas where access to dental care is limited (46).

The overall findings from this review compliment the WHO Global Oral Health Strategy 2022–2030, which promotes embedding oral health in primary care and noncommunicable disease (NCD) frameworks and calls for interprofessional collaboration and workforce innovation as enablers of universal health coverage (47). Similarly, the FDI World Dental Federation’s vision and advocacy, endorsed at the World Health Assembly, emphasised integrating oral health into broader health systems and NCD management (48). Evidence from comprehensive primary care and dental integration systematic reviews further corroborates our findings. For instance, Prasad and colleagues state “there are various ways of integration, such as interprofessional education, interprofessional collaborative practice, closed-loop referral process, and various public and private partnerships, and at the same time, there are a lot of barriers in integration” (49), Christian et al. (50) further add that “with governance and financing interventions are key drivers of the integration strategy, and more research and evaluation are required to identify best practice models of service integration”.

To the best of the authors’ knowledge, this is the first review to specifically explore the impact of integrating oral health within a cardiovascular disease (CVD) cohort, considering both patients and healthcare professionals. Another strength of this review is the inclusion of diverse designs (experimental, observational, mixed-methods, service evaluations), enabling a nuanced understanding of what works, for whom and in which settings.

Limitations of the evidence include heterogeneity of the outcomes and the study designs that precluded meta-analysis; short follow-up focused on process measures rather than long-term CVD endpoints; limited representation from low- and middle-income countries. Moreover, since this study included service evaluations, their quality assessment was conducted via case series appraisal tools which may not fully capture the contextual aspects of service evaluations, potentially biasing their quality assessments. Lastly, our review excluded grey literature, which may under-represent pragmatic program evaluations outside journals.

### 4.1 Implications

Findings from this review point to several practical and policy-level opportunities to strengthen oral–cardiac integration in Australia and countries with comparable health systems, for instance, medica-dental collaboration can be enhanced by embedding brief, validated oral health screening tools, such as the COH, mOAG or GOHAI into routine cardiac, stroke and primary care assessments, especially for those with complex needs. There should be a protocolised oral hygiene and education support for hospital and stroke rehabilitation clinics, documented and timely referral pathways to dental services is also important to improve collaborative care. Building workforce capability is equally important, this can include interprofessional education for nurses, allied health practitioners and medical staff, alongside the strategic use of tele-dentistry in regions with limited dental workforce capacity, would help normalise oral health as part of cardiovascular care.

Future studies should deploy well-powered RCTs and longitudinal cohorts with ≥24-month follow-up, standardised oral and CVD outcomes, economic evaluations and implementation science frameworks (fidelity, adoption, sustainability), to help increase investments in oral health care (46, 51). Trials should test multi-component bundles (education + screening + referral + periodontal therapy) to clarify dose–response and downstream cardio-metabolic impact.

At the system level, integrating oral health within broader non-communicable disease strategies and universal health coverage commitments is essential. This includes aligning national priorities with the *WHO Global Oral Health Action Plan 2023–2030*, particularly Strategy 4, which calls for “integrating essential oral health care into primary health care and ensuring financial protection and access to essential supplies” (47).

Achieving integration will require sustainable funding models that incentivise oral health activities delivered by non-dental professionals, as well as digital interoperability to support shared records and closed-loop referral pathways. In Australian context, subsidy schemes based on the fee-for-service payment models, directed at communities with complex and vulnerable needs, in remote and rural areas, can help ensure timely access to dental care (46).

Overall, there needs to be effort to make oral health equality central to the action plans, especially for rural, First Nations and low-income populations who face persistent barriers to oral health care.

## 5 Conclusion

The evidence mapped in this review shows that integrating oral health into cardiovascular care can be beneficial for promoting oral care access and positive oral hygiene behaviours, especially for those who are at risk of oral health associated NCDs. Incorporating brief screening, basic oral-hygiene support and clearer referral pathways into primary, cardiac and stroke services appear to help patients and gives clinicians greater confidence in addressing oral-systemic concerns. These improvements, however, remain largely confined to short-term oral health gains, due to limited evidence yet available on longer-term cardiovascular effects.

In order for implementing successful oral health integration, there is a need to shift from isolated, short-duration oral health focused initiatives towards sustained, well-designed programs, frameworks and even evaluation studies capable of demonstrating impact over time. In Australia, this also means addressing system-level constraints, such as fragmented funding arrangements and the continued exclusion of oral health from core chronic disease policy frameworks.

## Data Availability

Publicly available datasets were analyzed in this study. This data can be found at: all data analysed in this work are directly extracted from the reference list in this systematic review.
